# Dispositional mindfulness profiles and psychological symptoms: a latent profile analysis

**DOI:** 10.3389/fpsyg.2025.1411901

**Published:** 2025-01-29

**Authors:** Fereshteh Mehrabi, Shadi Beshai

**Affiliations:** ^1^Department of Psychology, University of Regina, Regina, SK, Canada; ^2^Department of Psychology, Concordia University, Montreal, QC, Canada; ^3^Department of Psychological Science, Kennesaw State University, Kennesaw, GA, United States

**Keywords:** dispositional mindfulness, latent profile analysis, depression, anxiety, Five-Facet Mindfulness Questionnaire

## Abstract

**Objectives:**

In this study, we aimed to (1) examine profiles of mindfulness using the short form of the FFMQ (FFMQ-SF), (2) identify the demographic predictor (i.e., sex) of mindfulness profile membership, and (3) examine associations of mindfulness profiles with psychological outcomes (i.e., anxiety and depressive symptoms).

**Methods:**

This cross-sectional study included 604 individuals recruited from Amazon’s Mechanical Turk platform (MTurk). We performed latent profile analyses (LPA) to explore the individual profiles based on scores on dispositional mindfulness facets. Dispositional mindfulness was measured using the Five-Facet Mindfulness Questionnaire-Short Form (FFMQ-SF). Depression was assessed using the Patient Health Questionnaire-9 (PHQ-9). Anxiety symptoms were measured by the Generalized Anxiety Disorder Scale (GAD-7).

**Results:**

We identified three mindfulness profiles including, Judgmentally Describing, Low Mindfulness, and Non-Judgmentally Describing. Participants in the Low Mindfulness group were more likely to be women compared to the other two profiles (Judgmentally Describing and Non-Judgmentally Describing groups). Participants in the Low Mindfulness group had the highest levels of anxiety and depressive symptoms, whereas individuals in the Non-Judgementally Describing group had the lowest levels of depression and anxiety.

**Conclusion:**

In the present study, we demonstrated three profiles of the FFMQ-SF, which had differential relationships with anxiety and depressive symptoms. Consideration of such profiles may help clinicians to develop more fine-tuned mindfulness-based psychological interventions.

## Introduction

Mindfulness refers to the awareness of moment-to-moment experiences with a non-judgmental and curious attitude (Kabat-Zinn and Hanh, 2009). Mindfulness has been conceptualized as a state (i.e., a momentary awareness of experiences) and a trait or disposition (i.e., a stable capacity to be aware of moment-to-moment experiences with non-judgment). Trait or dispositional mindfulness is a naturally occurring individual difference within the population, irrespective of mindfulness practice (Brown et al., 2007; Kabat-Zinn and Hanh, 2009; Tomlinson et al., 2018). Accordingly, people who are higher on dispositional mindfulness tend to experience more mindful states over time (Brown et al., 2007). There is also growing evidence showing that higher dispositional mindfulness is associated with better psychological health within the general population. For instance, evidence from multiple studies, systematic reviews and meta-analyses has demonstrated an inverse association between dispositional mindfulness and psychological disorder symptoms (e.g., depression and anxiety symptoms) as well as related outcomes such as general psychological distress, and negative or maladaptive cognitions (Keng et al., 2011; Vøllestad et al., 2012; Tomlinson et al., 2018; Juozelskyte and Catling, 2024).

It is essential to explore the granulated relationship between dispositional mindfulness and psychological outcomes due to its important implications for the potential management of health and the cultivation of well-being (Tomlinson et al., 2018). While several models of dispositional mindfulness have been proposed, the Five-Facet Mindfulness Questionnaire (FFMQ) is a widely acknowledged and supported conceptualization of dispositional mindfulness (Baer et al., 2006). This model suggests dispositional mindfulness is composed of five facets, including the ability to observe and describe internal and external experiences, act with awareness, focus on the present, the ability to be non-judging of internal and external experiences, and be non-reactive to these experiences (Baer et al., 2006). Evidence suggests that these facets and their unique combinations impact psychological outcomes in different ways. It is thus important to ascertain the details of the nature of the relationship between such facets and identify which facets positively influence psychological outcomes (Baer et al., 2006; Tomlinson et al., 2018).

Using multivariate regression analyses, researchers in a Swedish study, examined the relationships between the five facets of the FFMQ and psychological outcomes (i.e., depression, anxiety, stress, and positive states of mind) (Bränström et al., 2011). Bränström et al. (2011) demonstrated that acting with awareness and non-reactivity to experiences were strongly associated with psychological outcomes while the non-judgment facet was related only to anxiety. The ability to describe sensations, thoughts, and feelings—corresponding to the Describe facet of the FFMQ—was not related to symptoms of depression and anxiety (Bränström et al., 2011).

Prieto-Fidalgo et al. (2022) applied a meta-analytic structural equation modeling approach to explore the relationships between specific mindfulness facets and anxiety symptoms. They found that acting with awareness, non-judging, describing, and non-reacting were significantly associated with anxiety symptoms at baseline. However, only acting with awareness and non-reacting predicted reductions in anxiety symptoms over time, underscoring their prospective importance in improving mental health outcomes. A recent longitudinal study has shown that only three facets including acting with awareness, observing, and describing were associated with psychological symptoms over time (Cortazar and Calvete, 2019).

Although these studies have examined the associations between each facet of the FFMQ and psychological symptoms, they used variable-centered analyses (e.g., multiple regression). This approach is limited as it focuses on the unique associations between a single facet of mindfulness and related outcomes. A person-centered approach [i.e., Latent Profile Analysis (LPA)] can address this limitation by identifying people with distinct profiles of dispositional mindfulness. The person-centered approach emphasizes that facets of mindfulness are interconnected and individuals can differ in the combination of facets (Bravo et al., 2018; Calvete et al., 2020). In addition, the person-centered approach allows for a more nuanced understanding of mindfulness and its constituent parts, including the notion that different levels of each of the facets of the FFMQ can co-occur within the same individuals (Lam et al., 2018). Understanding how mindfulness profiles are related to prevalent symptoms of psychopathology is critical for tailoring mindfulness-based interventions to individuals based on their profiles to improve the quality and efficiency of mindfulness interventions (Bravo et al., 2018).

Previous research studies used LPA to identify subgroups of participants based on their scores on the five mindfulness facets. Several studies found four distinct profiles of FFMQ, including non-judgmentally aware, judgmentally observing, high mindfulness, and low mindfulness (Pearson et al., 2015; Bravo et al., 2016; Kimmes et al., 2017; Lam et al., 2018; Lubbers et al., 2024). Similarly, Gu et al. (2020) supported the presence of a four-profile solution, where three profiles—high mindfulness, nonjudgmentally aware, and low mindfulness—mapped broadly onto those identified in prior studies. However, the additional subgroup was the moderate mindfulness, not the judgmentally observing profile. De Souza Marcovski and Miller (2023) also identified three profiles consistent with prior studies: non-judgmentally aware, judgmentally observing, and high mindfulness. However, in contrast to earlier findings of a low mindfulness profile, they reported the presence of an average mindfulness profile, further diversifying our understanding of mindfulness profiles.

Although these studies replicated the four profiles in multiple populations, other research studies obtained different profiles. For example, Bravo et al. (2018) found three profiles (i.e., non-judgmentally aware, low, and high mindfulness profiles) on the same scale. Similar to the findings of Gu et al. (2020)’s study, Bravo et al. (2018) identified the high non-judgmentally aware and the high mindfulness profiles, but not the judgmentally observing profile. Likewise, Zhu et al. (2020) identified three profiles, including average mindfulness, low to average mindfulness, and high non-judgmentally aware. This study also identified the high non-judgmentally aware, but neither the high mindfulness profiles nor the judgmentally observing profiles. These discrepancies in results suggest that mindfulness profiles are different across samples and need further investigation. Additionally, the results of the above studies are mainly based on the combinations of homogeneous profiles (i.e., profiles that have consistent levels in mindfulness facets such as high or low mindfulness) and heterogeneous profiles (i.e., profiles that have simultaneously high and low levels of mindfulness facets such as Non-judgmentally Aware or Judgmentally Observing) and demonstrated instability in profile solution across studies (Lecuona et al., 2022).

Several of the above-mentioned studies have examined the association between the facets of mindfulness and different psychological outcomes. Pearson et al. (2015) found that the judgmentally observing and the low mindfulness groups had the poorest psychological outcomes (i.e., depressive symptoms, worry, and distress intolerance), whereas the high mindfulness and the nonjudgmentally aware groups had the greatest psychological outcomes. Bravo et al. (2016, 2018) demonstrated that the high mindfulness group had the highest levels of psychological outcomes (i.e., higher psychological flexibility, decentering, and self-regulation) compared to other profiles. Consistent with the previous studies (Pearson et al., 2015; Bravo et al., 2016; Bravo et al., 2018). Gu et al. (2020) found that the high mindfulness group had less depressive symptoms while the very low mindfulness group had more depressive symptoms. Zhu et al. (2020) found people with a high non-judgmentally aware profile reported better psychological outcomes compared to other profiles. The evidence so far suggests that high mindfulness is related to better psychological health while low mindfulness is related to poorer psychological health.

In addition to research showing that mindfulness profiles are associated with psychological outcomes, there is evidence that demographic factors are linked to mindfulness. Specifically, the findings of some research studies suggest that females may be more mindful than males. Research studies examining sex differences in specific mindfulness facets found that males had higher non-reactivity scores whereas females had higher scores on the observe facet than males (Bränström et al., 2011; Fogarty et al., 2015). As such, controlling for sex may be important when assessing the relationship between mindfulness and symptoms of psychological disorders.

In the present study, we aimed to extend prior research by applying person-centered analyses to the study of mindfulness among an online, crowdsourcing sample given that LPA on the profiles of the 15-item FFMQ among crowdsourcing online samples has not yet been done (Beshai et al., 2020), despite the increasing regularity with which these samples have been used in clinical research. Based on the person-centered approach, we used LPA to create classes or subgroups of participants based on their scores on each facet of FFMQ with others with similar patterns within this sample (Kimmes et al., 2017). The first objective of this study was to examine profiles of mindfulness using the short form of the FFMQ (FFMQ-SF) given that few research studies have examined the short form of mindfulness (Calvete et al., 2020; Lecuona et al., 2022). Second, we aimed to identify the demographic predictor (i.e., sex) of mindfulness profile membership. Third, we aimed to examine associations of mindfulness profiles with psychological outcomes (i.e., anxiety and depressive symptoms). Based on the results of the previous studies, we hypothesized that:

*H_1_*: Mindfulness profiles will demonstrate both homogeneous (similar across individuals) and heterogeneous (varied among individuals) patterns, reflecting the multifaceted nature of mindfulness.

*H_2_*: High mindfulness group would have the lowest level of anxiety and depressive symptoms.

## Methods

### Participants

For this cross-sectional study, we used the baseline data from a randomized controlled trial—the Mind-OP intervention—which was conducted from April to September 2019 (Beshai et al., 2020). A total of 604 individuals were recruited through Amazon’s Mechanical Turk, TurkPrime, an online crowdsourcing website (Litman et al., 2017) in April and May 2019. Less than half of the participants identified as women (41.9%). The mean age of the participants was 35.13 (SD = 10.57), and ages ranged between 19 and 72. Among the 604 participants, 86.1% (*n* = 522) had undergraduate education or below.

### Procedure

Participants completed study measures online, which were hosted on Qualtrics. Study measures were administered as part of the screening process for a randomized controlled trial assessing the effectiveness of an online mindfulness intervention (Beshai et al., 2020). Participants provided their informed consent and were then guided to complete study measures in randomized order. After completing the measures, participants completed a demographic information form, were thanked, and compensated with USD 2.5 for their participation. All participants received an initial general debrief; however, they were informed that a research assistant would contact them regarding their eligibility for the intervention trial. Ineligible participants were contacted within 7 days of completing screening measures and fully debriefed, while eligible participants were fully debriefed at the end of the trial period (Beshai et al., 2020).

### Measures

Dispositional mindfulness was measured with the Five-Facet Mindfulness Questionnaire-Short Form (FFMQ-SF-15) (Baer et al., 2008; Gu et al., 2016), which is derived from the original 39-item FFMQ (Baer et al., 2006). The five facets include (1) observing (e.g., “*I pay attention to sensations, such as the wind in my hair or sun on my face*”), (2) describing (e.g., “*I’m good at finding words to describe my feelings*”), (3) acting with awareness (e.g., “*I do jobs or tasks automatically without being aware of what I’m doing*”), (4) non-judging (e.g., “*I think some of my emotions are bad or inappropriate and I should not feel them*”), and (5) non-reactivity (e.g., “*When I have distressing thoughts or images I just notice them and let them go*”). The questionnaire contains 15 items scored on a 5-point Likert scale, ranging from 1 (*never or very rarely true*) to 5 (*very often or always true*). Higher scores indicated greater dispositional mindfulness. In the present sample, Cronbach’s alpha estimates for observing, describing, acting with awareness, non-judging, and non-reactivity facets were 0.74, 0.68, 0.70, 0.70, and 0.73, respectively.

Depressive symptoms were measured by the Patient Health Questionnaire-9 (PHQ-9) over the past 2 weeks (Spitzer et al., 2000). The PHQ-9 is a 9-item self-report depression scale measuring the presence and severity of Diagnostic and Statistical Manual (DSM) symptoms of major depressive disorder (e.g., sadness or low mood; anhedonia; sleep and appetite disturbance; psychomotor agitation or excitation over the last 2 weeks; fatigue; difficulty concentrating; and suicidal ideation). Scores for each item ranged from 0 (*not at all*) to 3 (*nearly every day*), with higher scores indicating more depressive symptoms. The Cronbach’s alpha internal consistency estimate for this scale was 0.92 in the current sample.

Anxiety symptoms were assessed using the Generalized Anxiety Disorder (GAD-7) (Spitzer et al., 2006) which assessed the presence and severity of anxiety symptoms in accordance with the DSM over the past week. The GAD-7 is a 7-item self-report questionnaire scored on a 4-point Likert scale, ranging from 1 (*not at all*) to 3 (*nearly every day*). Higher scores were indicative of greater distress. In the current sample, Cronbach’s alpha coefficient for this scale was 0.92.

### Data analyses

We applied the LPA (Ferguson et al., 2020) using the maximum likelihood estimation with robust standard errors (MLR) estimator in Mplus (version 8.8) (Muthén, 2021). The LPA was used to explore the individual profiles based on scores on the facets of the FFMQ-SF. We used a three-step BCH approach (Asparouhov and Muthén, 2014) which allows for the inclusion of covariates without altering profile composition. In step one, the number of latent profiles is estimated based on comparing the profile indicators, including Akaike Information Criterion (AIC), Bayesian Information Criterion (BIC), and sample-adjusted Bayesian Information Criterion (aBIC), the entropy index, and the Lo–Mendell–Rubin (LMR) tests. For AIC, BIC, and ABIC, smaller values predicted a better model fit. Entropy was used to assess the model’s accuracy within a range of 0 to 1; a higher score indicated a better model fit. The *p*-values of the LMR and Bootstrap Likelihood Ratio Tests (BLRT) were used to estimate whether a k class fits better than a k–1 class (Nylund-Gibson and Choi, 2018). In the second step, the latent class variable was formed based on the model-estimated probabilities of an individual belonging to each latent profile. In step three, we conducted multinomial logistic regression models using sex as a predictor of profile membership. In step four, the outcomes (depression and anxiety) were regressed on the latent class variable. We also tested for the equality of means across the classes on distal outcomes using the BCH method (Bolck et al., 2004) which uses posterior probability-based multiple imputations. This method accounts for the probabilistic nature of class membership and produces more unbiased standard errors than other methods (Asparouhov and Muthén, 2014).

## Results

### Participant characteristics

Descriptive statistics and correlations among the variables are shown in Table 1. Depression and anxiety symptoms were negatively correlated with scores on four of the FFMQ subscales except for observing. Among the five FFMQ subscales, acting with awareness was not correlated with observing. There were no correlations between non-judging and acting with awareness with non-reactivity. There was an inverse correlation between non-judging and observing.

**Table 1:** Descriptive statistics and correlations among study variables.

	1	2	3	4	5	6	7
1. Observing	1						
2. Describing	0.269^**^	1					
3. Acting with awareness	0.031	0.409^**^	1				
4. Non-judging	−0.088^*^	0.388^**^	0.531^**^	1			
5. Non-reactivity	0.315^**^	0.285^**^	0.41	0.036	1		
6. Depression (PHQ-9)	0.032	−0.325^**^	−0.533^**^	−0.604^**^	−0.175^**^	1	
7. Anxiety (GAD-7)	0.032	−0.335^**^	−0.579^**^	−0.631^**^	−0.109^**^	0.798^**^	1
Mean	9.67	10.01	10.26	10.26	8.72	7.72	8.75
SD	2.9	3	3	3.3	2.8	6	7

^**^Correlation is significant at the 0.01 level.

^*^Correlation is significant at the 0.05 level.

### Latent profile analysis

The indicators of the model fit for 1 through 5 class solutions are presented in Table 2. The LMR test indicates that the fit of the model with three profiles was significantly better (*p* < 0.001) than the model with two profiles, with no further improvement in fit when additional profiles are considered (0.0513). Further, the relative entropy of the 3-class solution (0.714) is considered high; whereas the relative entropy of the 2-class solution (0.607) did not approach a level of entropy that is considered high (Clark and Muthén, 2009). The AIC, BIC, and adjusted BIC decreased from a 1-class solution through a 5-class solution, indicating an improved fit as the number of class solutions increased, suggesting that a 3-class solution may be optimal. The results of the BLRT indicate that there were significant improvements in fit as each additional profile is added to the model (each *p* < 0.001), thus suggesting that five profiles be retained. However, we decided to retain three profiles because the BLRT can overestimate the number of profiles present (Morin and Marsh, 2015; Ferguson et al., 2020) and three profiles lend themselves to more meaningful interpretations (see Table 2).

**Table 2:** LPA model fit summary.

Fit indices	Number of profiles				
Fit statistics	1	2	3	4	5
Parameters	10	16	22	28	34
Log-likelihood	−3781.066	−3616.520	−3563.313	−3508.788	−3470.757
AIC	7582.131	7265.040	7170.626	7073.576	7009.514
BIC	7626.167	7335.497	7267.505	7196.876	7159.235
A-BIC	7594.419	7284.701	7197.660	7107.983	7051.294
Entropy	NA	0.676	0.714	0.724	0.779
Smallest class	NA	P 2 = 284 (47%)	P 2 = 55 (9%)	P 1 = 48 (8%)	P 5 = 57 (9%)
LMR test	NA	320.743	103.714	106.284	74.132
LMR *p*-value	NA	0.009	<0.001	0.0513	0.0422
BLRT test	NA	−3781.066	−3616.520	−3563.313	−3508.788
BLRT *p*-value	NA	<0.001	<0.001	<0.001	<0.001

*N = 604; AIC, Akaike Information Criterion; BIC, Bayesian Information Criterion; aBIC, Adjusted BIC; P, Profile; LMR, Lo–Mendell–Rubin likelihood ratio test; BLRT, Bootstrap Likelihood Ratio Test. The LMR test and the BLRT compare the fit of the model with k profiles to the adjacent model with k-1 profiles.*

**Figure 1:**
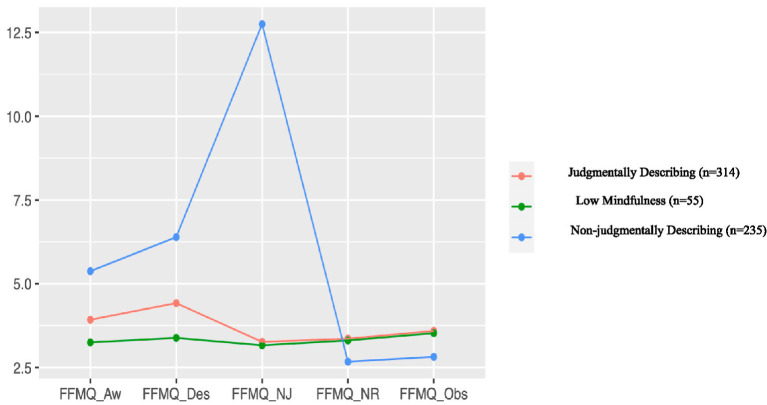
Latent profiles defined using the three facets of dispositional mindfulness (*n* = 604). FFMQ-Aw, mindfulness-acting with awareness; FFMQ-Des, mindfulness-describing; FFMQ-NJ, mindfulness-non judging; FFMQ-NR, mindfulness-non reactivity; FFMQ-Obs, mindfulness-observing.

Figure 1 depicts the pattern of means (standardized) across the 3 profiles. The first profile was the largest group and comprised 52% of the sample (*n* = 314). We labeled this profile the Judgmentally Describing group as this profile had low scores on observing, non-judgment, and non-reactivity facets and medium scores on awareness and describing facets of the FFMQ-SF. The second profile comprised 9% of the sample (*n* = 55). We labeled this profile the Low Mindfulness group as participants in this profile had low-to-average scores on every facet of the FFMQ-SF. The third profile had high scores on non-judgment and medium scores on awareness and describing, but very low scores in non-reactivity and observing facets. We labeled this class the Non-Judgmentally Describing group. Approximately, 39% of participants (*n* = 235) of the current sample were members of this profile.

### Multinomial regression results

#### Associations of mindfulness profiles with sex

Table 3 shows the associations between a predictor (sex) and the latent profiles, with the profile listed second in the heading serving as the reference group for each comparison. Participants in profile 3 (Low Mindfulness) were more likely to be women compared to profile 1 (Judgmentally Describing) and profile 2 (Non-Judgmentally Describing).

**Table 3:** Multinomial logistic regression results for the association between sex with three mindfulness profiles.

Covariate	Profile 2 vs. Profile 1 (ref)		Profile 3 vs. Profile 1 (ref)		Profile 1 vs. Profile 2 (ref)		Profile 3 vs. Profile 2 (ref)	
	OR	95%	OR	95%	OR	95%	OR	95%
Sex	0.744	0.480–1.151	0.301^**^	0.123–0.739	1.345	0.869–2.082	0.405^*^	0.173–0.952

^*^p < 0.05; ^**^p < 0.01. Profile 1, Judgmentally Describing; Profile 2, Non-Judgmentally Describing; Profile 3, Low Mindfulness.

#### Associations of mindfulness profiles with depression and anxiety

Mean comparisons are presented in Table 4. Superscripts indicate significant differences between profiles in each variable and are ordered in magnitude (details in Table 4). The low mindfulness profile displayed the highest levels of anxiety and depressive symptoms, while the Non-Judgementally Describing profile exhibited the lowest levels of depression and anxiety.

**Table 4:** Mean comparisons between latent profiles on mindfulness facets and psychological outcomes (depressive symptoms and anxiety).

	Judgmentally describing (*n* = 314)	Non-judgmentally describing (*n* = 235)	Low mindfulness (*n* = 55)
Mindfulness facets	M (SE)	M (SE)	M (SE)
Observing	2.718 (0.061)^3^	3.531 (0.074)^2^	4.381 (0.099)^1^
Describing	1.898 (0.045)^3^	3.239 (0.039)^1^	2.548 (0.061)^2^
Acting with awareness	2.236 (0.040)^2^	3.280 (0.039)^1^	1.444 (0.081)^3^
Non-judging	2.206 (0.049)^2^	3.331 (0.041)^1^	1.394 (0.070)^3^
Non-reactivity	2.331 (0.058)^3^	3.222 (0.068)^2^	4.346 (0.106)^1^
Psychological outcomes			
Anxiety	2.503 (0.095)^2^	0.881 (0.083)^3^	3.179 (0.227)^1^
Depressive symptoms	2.811 (0.111)^2^	0.888 (0.092)^3^	4.115 (0.284)^1^

Superscripts indicate mean differences between profiles (different superscripts = significant differences) and are ordered in magnitude. (^1^the profile with the highest value, and the others ranked in order).

The three profiles were statistically different from each other on all five facets of the FFMQ-SF. In the Non-Judgmentally Describing profile, the lowest average score was on observing and non-reactivity facets and the highest average score was in describing, acting with awareness, and non-judging facets. In other words, individuals in the Non-Judgmentally Describing profile were less likely to notice internal and external experiences. The Low Mindfulness and the Judgmentally Describing profiles had relatively little variation across the facets of the FFMQ-SF; however, the observing and non-reactivity facets exhibited the highest score in the Low Mindfulness profile (see Table 4).

## Discussion

In the current study, we investigated whether latent profiles of the FFMQ proposed in the literature were replicated using the short form of the FFMQ. In addition, we examined the relationships between mindfulness profiles with symptoms of anxiety and depression, considering sex as a covariate. Using LPA to evaluate profiles of dispositional mindfulness as measured by the FFMQ-SF-15, we identified three unique profiles. In line with the first hypothesis, we found a combination of homogenous and heterogenous mindfulness profiles. Specifically, we found one profile characterized by high non-judgment and describing (i.e., the non-judgmentally describing profile), one profile characterized by medium describing and low non-judgment (i.e., the judgmentally describing profile), and another one characterized by dispositional mindfulness traits that were lower than the sample’s mean (i.e., the low mindfulness profile). These results are inconsistent with the results of the previous studies that demonstrated the four-profile solution (Pearson et al., 2015; Bravo et al., 2016; Kimmes et al., 2017; Sahdra et al., 2017; De Souza Marcovski and Miller, 2023; Lubbers et al., 2024). However, the three-profile solution obtained in the current study is compatible with prior literature (Calvete et al., 2020; Lecuona et al., 2022; Echabe-Ecenarro et al., 2023).

The identified three profiles in the current study shared similarities on global levels of mindfulness (i.e., low, average, and high mindfulness) with the literature but differed on the configurations of mindfulness profiles (i.e., the non-judgmentally profile and the low profile). The general or low mindfulness profile was replicated in previous studies and was interpreted as homogeneous or sharing low scores across all facets (Zhu et al., 2020; Lecuona et al., 2022; Echabe-Ecenarro et al., 2023; Lubbers et al., 2024). Regarding the two heterogeneous profiles, previous research studies found a group showing average mindfulness with a judgmental observing profile and another group showing high levels of mindfulness with a high non-judgmentally aware profile (Calvete et al., 2020; Zhu et al., 2020; Lecuona et al., 2022; De Souza Marcovski and Miller, 2023; Lubbers et al., 2024).

Consistent with these studies, we found non-judgmental and high-judgmental profiles but with different constituent facets of mindfulness. The use of different versions of the FFMQ (short or full version), design and nature of studies, population characteristics, sample size, and recruitment populations could explain the discrepancies between studies. For example, given that the present study used the short version of the mindfulness scale (FFMQ-SF-15), some excluded items may better differentiate the high mindfulness profile from other profiles in terms of the non-judging facet (Lam et al., 2018). Future research studies in more homogenous samples and populations are warranted to explore this issue and discern the benefits of mindfulness programs among different groups.

The relationships between mindfulness facets and lower symptoms of depression and anxiety were consistent with the second hypothesis and existing literature. Specifically, our results illustrated that individuals with higher levels of mindfulness facets, particularly those in the Non-Judgementally Describing group, displayed significantly lower levels of anxiety and depressive symptoms. This pattern may be explained by improved cognitive and emotional processing, potentially facilitated by enhanced activation or connectivity in brain regions associated with emotional regulation, such as the prefrontal cortex (Keng et al., 2011). In contrast, individuals with lower levels of mindfulness facets demonstrated significantly greater levels of anxiety and depressive symptoms, likely attributable to a diminished capacity for effective emotional regulation and increased sensitivity to stressors (Shin et al., 2006). Notably, the small group of persons in the low mindfulness group reported the poorest outcomes, whereas most individuals in the non-judgmentally describing group achieved the best outcomes.

These findings align with prior research suggesting that mindfulness enhances cognitive flexibility and attentional functioning, both of which are key mechanisms for managing emotional responses and mitigating psychological distress (Moore and Malinowski, 2009; Hodgins and Adair, 2010; Keng et al., 2011; Im et al., 2021). While mindfulness practices such as Mindfulness-Based Stress Reduction (MBSR) and Mindfulness-Based Cognitive Therapy (MBCT) have been theorized to promote non-judgmental and non-reactive acceptance of experiences, our study emphasizes dispositional mindfulness—an inherent tendency to approach experiences mindfully (Gu et al., 2015; Im et al., 2021). Specifically, our findings support these theoretical underpinnings by showing that the ability to describe experiences non-judgmentally is particularly associated with improved emotional outcomes. Individuals in the Non-Judgementally Describing group exhibited significantly lower levels of anxiety and depressive symptoms compared to other groups, suggesting that this facet of mindfulness may reflect enhanced emotional regulation and a non-reactive approach to experiences, which are central to dispositional mindfulness.

Improvements in emotional regulation and attentional control are likely facilitated by the strengthening of neural pathways involved in these processes, which may explain the superior psychological outcomes observed in individuals with higher levels of mindfulness in this study. The positive effects of mindfulness facets, particularly in enhancing attention regulation and executive control, suggest that these inherent traits contributes to improved cognitive and emotional processing, ultimately fostering psychological well-being (Keng et al., 2011).

Neurobiological investigations provide additional insight into these mechanisms. Structural and functional changes in brain regions associated with attention and emotional regulation—such as increased cortical thickness in the prefrontal cortex and heightened activity in the rostral anterior cingulate cortex—have been observed in individuals with higher mindfulness levels (Lazar et al., 2005; Hölzel et al., 2011; Keng et al., 2011; Alexandra Kredlow et al., 2022). These findings underscore the potential of mindfulness, whether dispositional or cultivated through practice, to enhance resilience to psychological distress.

Overall, enhanced cognitive flexibility, attentional functioning, and emotional regulation may represent key mechanisms underlying the association between mindfulness facets and reduced symptoms of anxiety and depression. These processes, inherent in dispositional mindfulness, likely contribute to the psychological benefits observed in individuals with higher mindfulness levels, as demonstrated by the Non-Judgmentally Describing group in our study. These findings underscore the value of mindfulness facets as natural tendencies that promote emotional well-being and alleviate psychological distress.

In line with our results, previous research has demonstrated the relationship between mindfulness and psychological well-being. For example, Lecuona et al. (2022) and Lubbers et al. (2024) found that individuals with higher levels of mindfulness reported higher levels of psychological well-being, while people with lower levels of mindfulness were more prone to depression. Similarly, De Souza Marcovski and Miller (2023) observed that individuals in the high mindfulness group exhibited the lowest levels of depression and anxiety, whereas those in the judgmentally observing group showed the highest. Calvete et al. (2020) also illustrated that the judgmentally observing individuals experienced higher depressive symptoms, whereas the nonjudgmentally aware individuals had lower depressive symptoms, maladaptive schemas, and perceived stress. Zhu et al. (2020) and Echabe-Ecenarro et al. (2023) further confirmed that people in the high non-judgmentally aware group reported better psychological outcomes, followed by people in the average mindfulness group; however, people in low to average mindfulness reported poorer psychological outcomes. Additionally, Lam et al. (2018) demonstrated that low mindfulness and judgmentally observing profiles were associated with higher levels of depressive and anxious symptoms. Our findings added to the literature by providing evidence that the distinct profiles of trait mindfulness are related to both depression and anxiety symptoms.

In line with our findings, several research studies and interventions have demonstrated that higher levels of dispositional mindfulness were related to lower levels of stress, anxiety, and depression symptoms (Keng et al., 2011; de Bruin et al., 2012; Beshai et al., 2022; Prieto-Fidalgo et al., 2022). This evidence typically involves mindfulness-based practices that cultivate non-judgmental awareness of the present moment (Kabat-Zinn and Hanh, 2009; Gallego et al., 2014; Prieto-Fidalgo et al., 2022). Mindfulness can help individuals develop a non-reactive and accepting attitude toward their thoughts, negative emotions, and experiences and learn how to manage adverse emotional states and, particularly, stress which may ultimately lead to a reduction in rumination and negative thinking patterns associated with anxiety and depression symptoms. This increased self-awareness, acceptance, and attention to the present moment allow the emergence of any internal event and distance from those negative thoughts and emotions, leading to greater psychological flexibility (Langer et al., 2010; Gallego et al., 2014). Enhanced cognitive processes, such as improved attentional control, cognitive flexibility, and meta-cognition, are key mechanisms through which mindfulness achieves these outcomes. Mindfulness fosters the regulation of attention, enabling individuals to interrupt maladaptive cognitive patterns like rumination, which often sustain anxiety and depression. This supports the idea that those who are not judgmental and critical toward their internal experiences (Non-Judgementally Describing group) exhibited less anxiety and depressive symptoms in the present study.

Regarding sex, as a predictor of the three-profile model, significant differences emerged only among participants in the low mindfulness group. We found that people in the low mindfulness profile were more likely to be women than each of the other two profiles. These findings are generally in line with previous studies. Zhu et al. (2020) found that people in the high non-judgmentally aware group with a high level of global mindfulness were more likely to be male than people in average mindfulness and low to average mindfulness groups. Likewise, Lam et al. (2018) found that sex was significantly associated with the latent profiles of mindfulness among cancer patients. Patients who were male were significantly more likely to demonstrate non-judgmentally aware as compared to the high mindfulness profile. Sahdra et al. (2017) found that younger male participants were more likely than females to belong to the judgmentally observing group.

### Strengths, limitations, and future research

The main strength of this study is that this is the first study that focuses on the FFMQ-SF-15 with a crowdsourcing online sample. This focus is particularly important given that each version of the FFMQ scale found different facets of mindfulness predict different psychological outcomes. However, while the use of the FFMQ-SF is validated and efficient for online research, it presents certain limitations. The decision to use the FFMQ-SF in this study was driven by the need to balance participant burden with data quality and reliability (Gu et al., 2016; Shallcross et al., 2020).

As a shortened version of the original 39-item FFMQ, the FFMQ-SF may provide less detailed information about each mindfulness facet, potentially constraining the study’s ability to fully capture the complexity of mindfulness profiles. Understanding which items yield the most information is particularly valuable, as the full 39-item FFMQ can be challenging to implement in studies where participant burden is a concern. A streamlined version of the FFMQ that retains the most informative items while excluding less valuable ones could offer an efficient yet comprehensive measure of mindfulness (Shallcross et al., 2020).

In future research, employing the full FFMQ could provide a more nuanced understanding of mindfulness profiles and their relationships with psychological outcomes, potentially revealing additional profiles that were not captured in this study. Additionally, future research could also explore the extent to which the profiles identified in this study remain consistent across different measures of mindfulness. Such efforts could further validate the identified profiles and ensure their generalizability across diverse contexts and populations.

Finally, the borderline reliability of the FFMQ-15 suggests that clinical implications derived from these findings should be interpreted with caution until future research using more comprehensive measures and more recent data replicates these results.

One additional limitation to consider is the cross-sectional nature of our data, which precludes any conclusion regarding causal relationships and longitudinal patterns. To address this limitation, future research should employ longitudinal designs to gain a deeper understanding of how mindfulness evolves over time. Latent transition analysis (LTA) could be used to examine how individuals transition between different mindfulness profiles, tracking shifts in latent class membership over time and providing insights into the dynamic relationship between mindfulness and mental health outcomes. Furthermore, longitudinal latent class analysis (LLCA) could offer a more nuanced understanding of the heterogeneity in mindfulness profiles. LLCA captures changes in latent class membership across repeated measures, offering valuable insights into patterns of change (Nylund-Gibson and Choi, 2018).

Another limitation is that all instruments were self-reported scales which may have inherent limitations, such as susceptibility to social desirability bias or potential inaccuracies in self-perception. These limitations are particularly relevant when assessing subjective constructs like mindfulness and psychological symptoms. The validity of such measures falls short in their assessment of psychological disorders compared with the gold-standard structured clinical interviews. To address this limitation, future research could incorporate objective measures, such as physiological indicators like salivary cortisol levels to assess stress or heart rate variability for anxiety (Keng et al., 2011; Hoge et al., 2019). By combining self-reports with these objective measures, researchers can enhance the reliability of findings and gain a more comprehensive understanding of changes in emotion-related outcomes. This multi-method approach would help confirm self-reported symptoms and mindfulness levels, and continue to advance the accuracy and robustness of emotional and psychological assessments (Hoge et al., 2019).

Another limitation is that participant recruitment relied on Amazon Mechanical Turk (MTurk), which is known for its demographic diversity compared to traditional Internet and college samples. However, MTurk participants often share certain characteristics, such as higher technological proficiency and specific socio-demographic profiles, which may limit the generalizability of the findings. Although MTurk currently provides access to a diverse participant pool, changes in its platform dynamics over time could affect the representativeness of samples. To enhance external validity and achieve a more comprehensive representation of mindfulness profiles in the general population, future research should diversify recruitment efforts to include participants from sources such as universities and clinical settings (Buhrmester et al., 2011). An experimental approach, where mindfulness profiles are assessed before and after a mindfulness intervention, would allow for comparison of specific effects within each profile, adding practical applicability. This could help identify how different profiles respond to mindfulness training and whether certain profiles benefit more from specific types of interventions, ultimately guiding tailored therapeutic approaches.

Lastly, we did not consider the overall level of high/low responses on subscales by participants. It is important to distinguish between the level effect or quantitative aspect (i.e., the overall low, medium, or high levels across all mindfulness factors) and shape effect or qualitative aspect (i.e., specific patterns of high, medium, or low levels of factors) in the mindfulness profiles. Future studies should separate the extent to which individuals generally report high mindfulness across all facets (the level effect) from the extent to which these individuals are relatively more mindful on some facets than others (the shape effect) (Sahdra et al., 2017; Bravo et al., 2018).

### Implications for clinical practice

Our findings suggest that mindfulness-based interventions can be tailored to more specifically target patients in each of the three derived latent profiles. People in the low general mindfulness group may benefit from general mindfulness training to target the cultivation of all facets of the construct. This would be contrasted to training for individuals displaying the Non-Judgementally Describing profile, who may benefit more from interventions that specifically cultivate the non-reactivity component of mindfulness. Accordingly, tailoring or adaptation of mindfulness-based interventions can be informed by considering the unique combinations of facets of the FFMQ and the differential relationships of such combinations with symptoms of depression and anxiety among high-risk populations. In particular, person-centered approaches may help in the development of cost-effective mindfulness-based practices and be an important step toward maximizing outcomes of mindfulness-based interventions in clinical practices (Kimmes et al., 2017). Further, our findings, especially once replicated, suggest a sex-sensitive approach to deploying mindfulness training. Mindfulness training may be incorporated into interventions for conditions that disproportionately affect women (e.g., eating disorders). Moreover, our findings suggest clinicians should pay particular attention to general mindfulness profiles of women seeking mental health care. Health promotion interventions should focus on promoting mindfulness skills, mindsets, and emotion regulation strategies to alleviate depressive and anxiety symptoms among at-risk populations.

## Conclusion

In this study, we demonstrated a three-profile solution describing self-reported dispositional mindfulness scores on the FFMQ-SF. The three-profile solution consisted of Non-Judgementally Describe, Judgementally-Describe, and Low Mindfulness profiles, with participants in the Non-Judgementally Describe profile demonstrating the lowest symptoms of psychopathology. Given that we found a link between psychological disorder symptoms and dispositional mindfulness class membership, future studies can explore how such profiles may associate with other key factors in psychopathology (i.e., cognitive processes, emotional regulation) (Tomlinson et al., 2018) or how to target the cultivation of specific profiles using mindfulness practice.

## Data Availability

The data analyzed in this study is subject to the following licenses/restrictions: the data will be available from the second author (SHB) upon reasonable request. Requests to access these datasets should be directed to sbeshai@kennesaw.edu.
